# Editorial: Psychological factors as determinants of medical conditions, volume III

**DOI:** 10.3389/fpsyg.2026.1867939

**Published:** 2026-06-09

**Authors:** Gabriella Martino, Concetto Mario Giorgianni, Peter Schwarz, Carmelo Mario Vicario

**Affiliations:** 1 Department of Clinical and Experimental Medicine, University of Messina, Messina, Italy; 2 Department of Biomedical and Dental Sciences and Morphofunctional Imaging, University of Messina, Messina, Italy; 3 Department of Endocrinology, Research Centre for Ageing and Osteoporosis, Rigshospitalet-Glostrup Hospital, Copenhagen, Denmark; 4 Department of Cognitive Sciences, Psychology, Education and Cultural Studies, University of Messina, Messina, Italy

**Keywords:** adherence, chronic diseases, emotional distress, psychological factors, quality of life

Life expectancy is steadily increasing worldwide, and age-related diseases continue to represent a major challenge for contemporary healthcare systems. Chronic medical conditions, including metabolic, endocrine, cardiovascular, and inflammatory diseases, exert a profound impact not only on physical health but also on individuals' perceived quality of life. This complex interplay frequently gives rise to psychopathological symptoms, which are now widely recognized as closely intertwined with the course and outcomes of medical illness. Within this perspective, psychological factors do not merely accompany chronic diseases but actively contribute to their development, progression, and management (Kiecolt-Glaser et al., 2002; Castelnuovo et al., 2015).

Building upon the foundations laid by the previous volumes of this Research Topic, the present Volume III aims to further advance the understanding of the complex interrelations between psychological processes and medical conditions. While the first volume primarily highlighted the relevance of psychological determinants in medical settings (Martino et al., 2019), and the second volume emphasized their multidimensional and methodological articulation (Caputo et al., 2022), this third installment moves toward a more integrated and process-oriented perspective. Specifically, it underscores the role of psychological factors as dynamic processes that shape individual health trajectories over time, influencing both subjective experience and objective clinical outcomes.

A growing body of evidence suggests that psychological processes, such as emotional regulation, cognitive functioning, personality organization, and health-related behaviors, operate in a deeply interconnected manner (Castelnuovo, 2010; Conversano, 2019; Conversano and Di Giuseppe, 2021; Di Giuseppe et al., 2020; Vicario and Martino, 2025). These processes influence how individuals perceive illness, adhere to medical recommendations, and engage in health-promoting or health-compromising behaviors. In this sense, the traditional body-mind dichotomy is increasingly replaced by a bidirectional and dynamic model in which psychological and somatic dimensions continuously interact.

The contributions included in this Research Topic reflect this evolving framework and can be organized into several interrelated domains.

A first cluster of studies focuses on emotional processing, psychological distress, and adaptation to illness. These contributions explore the role of anxiety, depression, illness perception, trauma-related experiences, and quality of life across a wide range of clinical conditions, including cancer, multiple sclerosis, cardiovascular disease, and perinatal contexts (Szablewska et al.; Lei et al.; Yang et al.; Gómez-Ballesteros et al.; Huang et al.; Kandasamy et al.; Li et al.; Yun et al.; Aghamohammadi et al.). Emotional factors emerge as central components in shaping patients' experiences of illness and their engagement with treatment, confirming their pivotal role in both clinical expression and disease management, particularly in relation to endocrine conditions and the interplay between thyroid function and depression (Jørgensen and Feldt-Rasmussen, 2018; Martino et al.), as well as in disorders such as Hashimoto's thyroiditis, where emotional distress and quality of life are closely intertwined (Martino et al., 2021b), and more broadly in endocrine conditions such as hyperthyroidism, where quality of life represents a key clinical outcome (Vita et al., 2020). In line with these findings, increasing evidence highlights the role of alexithymia and emotional dysregulation in immune-mediated and chronic conditions, including asthma (Silvestro et al., 2023; Ricciardi et al., 2023) and inflammatory bowel diseases (Martino et al., 2020b), as well as in other chronic conditions such as cancer, where psychological distress and defense styles play a crucial role (Conversano et al., 2020; Di Giuseppe et al., 2019).

A second group of studies investigates personality-related dimensions and underlying psychological mechanisms, including defense mechanisms, epistemic stances, and emotional intelligence (Martino et al.; Di Giuseppe et al.; Cruciani et al.; Suslow et al.). These contributions highlight the relevance of implicit and structural aspects of psychological functioning, suggesting that individual differences in personality organization and defensive processes may significantly influence both vulnerability to disease and adaptive responses to chronic conditions.

A third domain encompasses health-related behaviors and adherence to medical recommendations. Studies addressing vaccination hesitancy, treatment adherence, illness perception, and behavior under health-related constraints emphasize the critical role of psychological variables in guiding decision-making processes and shaping health behaviors (Demir Pervane et al.; Lei et al.; Gómez-Ballesteros et al.; Yun et al.). In this perspective, underlying psychological processes such as defense mechanisms have been shown to significantly influence adherence and perceived quality of life in chronic conditions such as diabetes (Marchini et al., 2021; Martino et al., 2020a).

These findings reinforce the notion that effective medical management requires a comprehensive understanding of patients' psychological profiles and motivational dynamics.

A fourth cluster is represented by intervention-based studies, which examine the effectiveness of psychological and behavioral approaches in improving clinical outcomes. Contributions focusing on positive psychology interventions, group-based mental health education, physical exercise, and neuromodulation techniques demonstrate that targeting psychological processes can lead to meaningful improvements in both mental and physical health (Qian et al.; Wang et al.; Tang et al.; Radin et al.).

Finally, a fifth group of contributions advances integrative and methodological perspectives, proposing novel frameworks for understanding the relationship between psychological factors and medical conditions. These include systems-level analyses of cyberchondria in the digital age, highlighting how digital environments may amplify health-related anxiety through complex interactions between information exposure and individual vulnerability (Martino et al.). They also encompass bioethical reflections in clinical settings, investigations of gut microbiota in depression, and innovative modeling approaches to affect dynamics (Giacobello; Zhang et al.; Borghesi et al.), as well as emerging research on the role of artificial intelligence in shaping the doctor-patient relationship (Merlo et al., 2026).

Together, these studies underscore the need for increasingly sophisticated theoretical and methodological tools capable of capturing the complexity of mind-body interactions, in line with emerging perspectives on dynamic and non-linear systems in health research, with particular relevance for perceived quality of life across different clinical populations, including patients with severe hypersensitivity reactions (Ricciardi et al., 2024).

Similarly, in conditions such as osteoporosis, psychological factors, including anxiety and broader clinical-psychological features, have been shown to influence objective health outcomes such as bone mineral density (Martino et al., 2021a, 2023).

Taken together, the studies included in this volume reinforce the notion that psychological determinants of medical conditions should be conceptualized as dynamic, multilevel processes rather than isolated variables. Emotional, cognitive, and behavioral dimensions converge in shaping individual health trajectories, suggesting that an integrated and process-based approach is essential for both research and clinical practice.

From a clinical perspective, these findings highlight the importance of incorporating psychological assessment and intervention into routine medical care. From a research standpoint, they call for the development of longitudinal and process-oriented models capable of capturing the evolving nature of psychological and somatic interactions. Ultimately, this integrated perspective may contribute to the development of more personalized and effective strategies for prevention, diagnosis, and treatment.

It has been a great pleasure and honor to contribute to this Research Topic. We would like to sincerely thank all Authors for their valuable contributions, the Reviewers for their expertise and dedication, and the Frontiers Editorial and Developmental Staff for their continuous support. The richness and diversity of the contributions collected in this third volume not only consolidates the scientific relevance of this Research Topic but also opens promising directions for future research. In this regard, we look forward with great interest to further developments in this field, as the ongoing evolution of this series continues to deepen our understanding of the complex interplay between psychological factors and medical conditions.

## References

[B1] Caputo A. Vicario C. M. Cazzato V. Martino G. (2022). Editorial: Psychological factors as determinants of medical conditions, volume II. Front. Psychol. 13:865235. doi: 10.3389/fpsyg.2022.865235 35386893 PMC8977586

[B2] Castelnuovo G. (2010). No medicine without psychology: the key role of psychological contribution in clinical settings. Front. Psychol. 1:4. doi: 10.3389/fpsyg.2010.00004 21833187 PMC3153736

[B3] Castelnuovo G. Pietrabissa G. Manzoni G. M. Corti S. Ceccarini M. Borrello M. . (2015). Chronic care management of globesity: promoting healthier lifestyles in traditional and mHealth based settings. Front. Psychol. 6:1557. doi: 10.3389/fpsyg.2015.01557 26528215 PMC4606044

[B4] Conversano C. (2019). Common psychological factors in chronic diseases. Front. Psychol. 10:2727. doi: 10.3389/fpsyg.2019.02727 31866912 PMC6909152

[B5] Conversano C. Di Giuseppe M. (2021). Psychological factors as determinants of chronic conditions: clinical and psychodynamic advances. Front. Psychol. 12:635708. doi: 10.3389/fpsyg.2021.635708 33584488 PMC7876054

[B6] Conversano C. Di Giuseppe M. Miccoli M. Ciacchini R. Di Silvestre A. Lo Sterzo R. . (2020). Retrospective analyses of psychological distress and defense style among cancer patients. Clin. Neuropsychiatry 17, 217–224. doi: 10.36131/cnfioritieditore20200403 34908997 PMC8629055

[B7] Di Giuseppe M. Di Silvestre A. Lo Sterzo R. Hitchcott P. Gemignani A. Conversano C. (2019). Qualitative and quantitative analysis of the defensive profile in breast cancer women: a pilot study. Health Psychol. Open 6:2055102919854667. doi: 10.1177/2055102919854667 31218073 PMC6558547

[B8] Di Giuseppe M. Perry J. C. Conversano C. Gelo O. C. G. Gennaro A. (2020). Defense mechanisms, gender, and adaptiveness in emerging personality disorders in adolescent outpatients. J. Nerv. Ment. Dis. 208, 933–941. doi: 10.1097/NMD.0000000000001230 32947450

[B9] Jørgensen M. B. Feldt-Rasmussen U. (2018). “Thyroid function and depression,” in Encyclopedia of Endocrine Diseases, 2nd Edn. (Academic Press), 792–801. doi: 10.1016/B978-0-12-801238-3.96001-X

[B10] Kiecolt-Glaser J. K. McGuire L. Robles T. F. Glaser R. (2002). Emotions, morbidity, and mortality: new perspectives from psychoneuroimmunology. Annu. Rev. Psychol. 53, 83–107. doi: 10.1146/annurev.psych.53.100901.135217 11752480

[B11] Marchini F. Langher V. Napoli A. Balonan J. T. Fedele F. Martino G. . (2021). Unconscious loss processing in diabetes: associations with medication adherence and quality of care. Psychoanal. Psychother. 35, 5–23. doi: 10.1080/02668734.2021.1922492

[B12] Martino G. Bellone F. Vicario C. M. Gaudio A. Caputo A. Corica F. . (2021a). Anxiety levels predict bone mineral density in postmenopausal women undergoing oral bisphosphonates: a two-year follow-up. Int. J. Environ. Res. Public Health 18:8144. doi: 10.3390/ijerph18158144 34360437 PMC8346074

[B13] Martino G. Bellone F. Vicario C. M. Gaudio A. Corica F. Squadrito G. . (2023). Interrelations between clinical-psychological features and bone mineral density changes in post-menopausal women undergoing anti-osteoporotic treatment: a two-year follow-up. Front. Endocrinol. 14:1151199. doi: 10.3389/fendo.2023.1151199 PMC10203700 37229451

[B14] Martino G. Caputo A. Bellone F. Quattropani M. C. Vicario C. M. (2020a). Going beyond the visible in type 2 diabetes mellitus: defense mechanisms and their associations with depression and health-related quality of life. Front. Psychol. 11:267. doi: 10.3389/fpsyg.2020.00267 32174865 PMC7054284

[B15] Martino G. Caputo A. Schwarz P. Fries W. Bellone F. Quattropani M. C. . (2020b). Alexithymia and inflammatory bowel disease: a systematic review. Front. Psychol. 11:1763. doi: 10.3389/fpsyg.2020.01763 32973596 PMC7466427

[B16] Martino G. Caputo A. Vicario C. M. Feldt-Rasmussen U. Watt T. Quattropani M. C. . (2021b). Alexithymia, emotional distress and perceived quality of life in patients with Hashimoto's thyroiditis. Front. Psychol. 12:667237. doi: 10.3389/fpsyg.2021.667237 34045997 PMC8144453

[B17] Martino G. Langher V. Cazzato V. Vicario C. M. (2019). Editorial: Psychological factors as determinants of medical conditions. Front. Psychol. 10:2502. doi: 10.3389/fpsyg.2019.02502 31781002 PMC6857525

[B18] Merlo E. M. Sparacino G. Silvestro O. Giacobello M. L. Meduri A. Casciaro M. . (2026). The role of artificial intelligence in shaping the doctor–patient relationship: a narrative review. Healthcare 14:481. doi: 10.3390/healthcare14040481 41753994 PMC12941156

[B19] Ricciardi L. Silvestro O. Martino G. Catalano A. Vicario C. M. Lund-Jacobsen T. . (2024). Health-related quality of life in severe hypersensitivity reactions: focus on severe allergic asthma and hymenoptera venom anaphylaxis: a cross-sectional study. Front. Psychol. 15:1394954. doi: 10.3389/fpsyg.2024.1394954 39246313 PMC11377323

[B20] Ricciardi L. Spatari G. Vicario C. M. Liotta M. Cazzato V. Gangemi S. . (2023). Clinical psychology and clinical immunology: is there a link between alexithymia and severe asthma? Mediterr. J. Clin. Psychol. 11, 1–18. doi: 10.13129/2282-1619/mjcp-3704

[B21] Silvestro O. Ricciardi L. Catalano A. Vicario C. M. Tomaiuolo F. Squadrito G. . (2023). Alexithymia and asthma: a systematic review. Front. Psychol. 14:1221648. doi: 10.3389/fpsyg.2023.1221648 37609491 PMC10441120

[B22] Vicario C. M. Martino G. (2025). Linkage among cognition, emotion and behavior: an interdisciplinary perspective. Brain Sci. 15:962. doi: 10.3390/brainsci15090962 41008322 PMC12469241

[B23] Vita R. Caputo A. Quattropani M. C. Watt T. Feldt-Rasmussen U. Puleio P. . (2020). Quality of life in patients with hyperthyroidism: where do we stand? Mediterr. Mediterr. J. Clin. Psychol. 8:2521. doi: 10.6092/2282-1619/mjcp-2521

